# PLEKHA7, an Apical Adherens Junction Protein, Suppresses Inflammatory Breast Cancer in the Context of High E-Cadherin and p120-Catenin Expression

**DOI:** 10.3390/ijms22031275

**Published:** 2021-01-28

**Authors:** Lindy J. Pence, Antonis Kourtidis, Ryan W. Feathers, Mary T. Haddad, Sotiris Sotiriou, Paul A. Decker, Aziza Nassar, Idris T. Ocal, Sejal S. Shah, Panos Z. Anastasiadis

**Affiliations:** 1 Department of Cancer Biology, Mayo Clinic, Jacksonville, FL 32224, USA; pence.lindy@mayo.edu (L.J.P.); feathers.ryan@mayo.edu (R.W.F.); haddad.mary@mayo.edu (M.T.H.); 2 Department of Regenerative Medicine and Cell Biology, Medical University of South Carolina, Charleston, SC 29425, USA; kourtidi@musc.edu; 3 Department of Laboratory Medicine and Pathology, Mayo Clinic, Rochester, MN 55901, USA; sotiriou.sotiris@mayo.edu; 4 Department of Health Sciences Research, Mayo Clinic, Rochester, MN 55901, USA; decker.paul@mayo.edu; 5 Department of Laboratory Medicine and Pathology, Mayo Clinic, Jacksonville, FL 32224, USA; nassar.aziza@mayo.edu; 6 Department of Laboratory Medicine and Pathology, Mayo Clinic, Phoenix, AZ 85054, USA; Ocal.Tolgay@mayo.edu; 7 Department of Pathology, Kaiser Permanente, Irvine, CA 92618, USA; sejal.s.shah@kp.org

**Keywords:** Inflammatory breast cancer, PLEKHA7, adherens junction, cadherin–catenin complexes

## Abstract

Inflammatory breast cancer is a highly aggressive form of breast cancer that forms clusters of tumor emboli in dermal lymphatics and readily metastasizes. These cancers express high levels of E-cadherin, the major mediator of adherens junctions, which enhances formation of tumor emboli. Previous studies suggest that E-cadherin promotes cancer when the balance between apical and basolateral cadherin complexes is disrupted. Here, we used immunohistochemistry of inflammatory breast cancer patient samples and analysis of cell lines to determine the expression of PLEKHA7, an apical adherens junction protein. We used viral transduction to re-express PLEKHA7 in inflammatory breast cancer cells and examined their aggressiveness in 2D and 3D cultures and in vivo. We determined that PLEKHA7 was deregulated in inflammatory breast cancer, demonstrating improper localization or lost expression in most patient samples and very low expression in cell lines. Re-expressing PLEKHA7 suppressed proliferation, anchorage independent growth, spheroid viability, and tumor growth in vivo. The data indicate that PLEKHA7 is frequently deregulated and acts to suppress inflammatory breast cancer. The data also promote the need for future inquiry into the imbalance between apical and basolateral cadherin complexes as driving forces in inflammatory breast cancer.

## 1. Introduction

Inflammatory breast cancer (IBC) is an aggressive subset of breast cancer, comprising approximately 2% of breast cancer diagnoses in the US [1,2]. Yet mortality from IBC is disproportionately responsible for around 7% of deaths from breast cancer each year [1]. IBC requires both clinical and pathologic diagnoses. Patients develop rapid onset (<6 months) redness, painful swelling, and dimpling of the skin, referred to as “peau-de-orange” because the skin resembles that of an orange peel, encompassing at least one-third of the breast. In addition to this clinical picture, IBC requires pathologic diagnosis of invasive carcinoma [3]. Dermal biopsy frequently reveals the presence of “tumor emboli” in the dermal lymphatics. The characteristic tumor emboli are considered responsible for the clinical phenotype of IBC patients, as these emboli may clog the lymphatic drainage system.

Extensive work has been done to profile IBC patient samples by RNA and genetics in order to understand the differences between IBC and non-IBC. This has proved quite challenging (see [4] for a review). However, over 20 years ago, it was observed that IBC patients and IBC models express disproportionately high levels of the adherens junction (AJ) protein E-cadherin [5,6,7]. Targeting E-cadherin with function-blocking antibodies in the MARY-X animal model of IBC led to a dramatic reduction in the number and size of tumor emboli [7]. It is expected that E-cadherin maintains adhesion between tumor cells within the emboli to facilitate safe and effective passage through the lymphatics. The viability of tumor emboli within circulatory systems likely contributes to high rates of metastasis in IBC.

E-cadherin mediates epithelial cell–cell adhesion via the trans-homophilic interaction of neighboring cells with E-cadherin. It binds several catenin family members via its cytoplasmic domain and is linked to the actin and microtubule cytoskeleton through these interactions [8]. E-cadherin is regularly turned over via endosome-lysosomal sorting but this turnover is inhibited by E-cadherin interaction with p120-catenin (p120) [9,10,11]. p120 binds the cytoplasmic juxtamembrane region of E-cadherin and protects E-cadherin from cleavage, clathrin-mediated endocytosis or ubiquitination by Hakai [12,13,14,15,16]. Not surprisingly, p120 is also required for IBC tumor growth and emboli formation. p120 expression is increased in IBC through internal ribosomal entry site (IRES)-mediated translation via the translation initiation factor eIF4GI, which is overexpressed in approximately 80% of IBC patient samples [17].

The cadherin–catenin complexes localize at the apical AJs and also along basolateral contacts [18]. Recent work from our lab determined that the basolateral complexes promote tumor progression via increased expression of Cyclin D1, Snail, and Myc and also increased Src family kinase activity [19]. However, apical cadherin–catenin complexes suppress the translation of these proteins through the function of PLEKHA7, an apical AJ-specific interacting partner of p120 [19,20,21]. PLEKHA7 recruits the micro-RNA (miRNA) processing machinery to the apical AJs, promotes the generation of mature miRNAs, and facilitates loading of miRNAs onto a junctional RNA-induced silencing complex (RISC), resulting in local suppression of mRNA translation [19,22]. Loss of PLEKHA7-mediated RNA interference (RNAi) at the apical AJs leads to higher expression of Cyclin D1, Snail, and Myc and results in gain of anchorage independent growth (AIG) in colon epithelial cells [19]. 

Given that IBC patients express high levels of E-cadherin and p120, we hypothesized that an imbalance of the pro-growth basolateral cadherin–catenin complexes and tumor-suppressing apical AJs contributes to the pathophysiology of IBC. In this study, we looked at PLEKHA7 expression and function in IBC models. We found that patients and cell models largely lack functional PLEKHA7. When PLEKHA7 was restored in cell and xenograft models, we found that the aggressive nature of IBC was mitigated. Our results provide insight into the nuances of the cadherin–catenin axis’s contribution to IBC and suggest that PLEKHA7 acts as a suppressor of IBC.

## 2. Results

**Figure 1:**
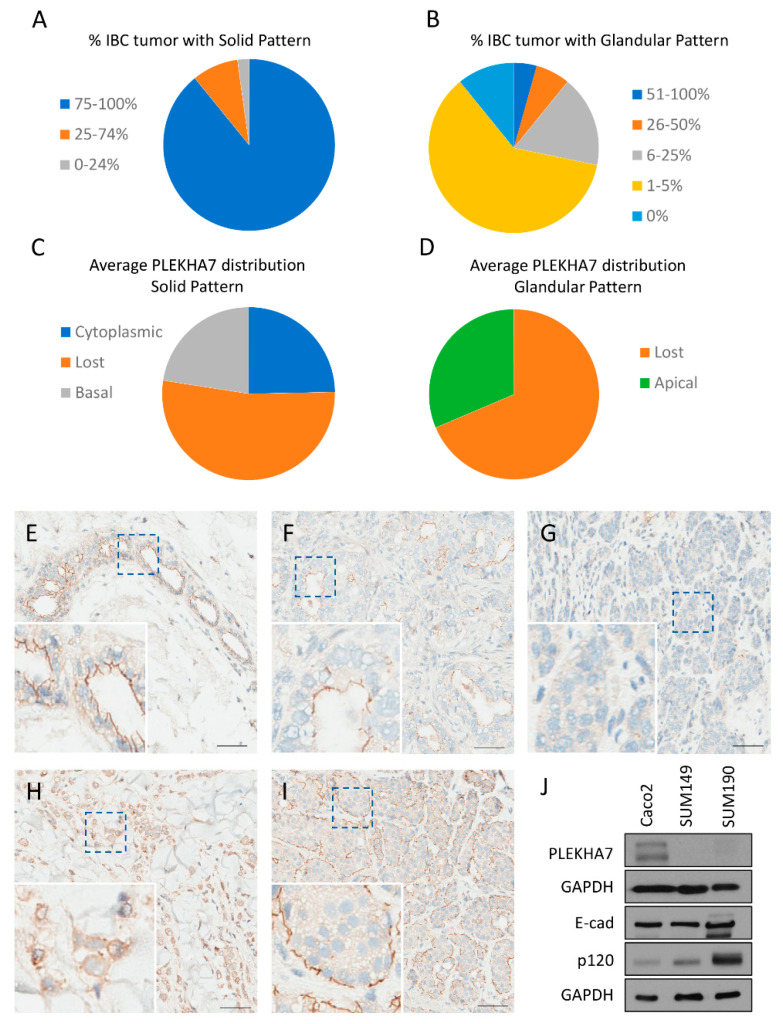
PLEKHA7 expression in inflammatory breast cancer (IBC) patient samples and cell lines. (**A**) Pie chart displaying the number of IBC patient samples demonstrating solid tumor patterns in 0–24% (N = 1 tumor), 25–74% (N = 4 tumors), or 75–100% (N = 41 tumors) of the total tumor. (**B**) Pie chart displaying the number of IBC patient samples demonstrating glandular tumor patterns in 0% (N = 5 tumors), 1–5% (N = 28 tumors), 6–25% (N = 8 tumors), 26–50% (N = 3 tumors), or 51–100% (N = 2 tumors) of the total tumor. (**C**) Pie chart depicting the average percentage of PLEKHA7 expression as lost (56.4%), cytoplasmic (26.4%), or basal (24.1%) in the regions of solid tumor from all IBC patient samples. No apical staining of PLEKHA7 was observed in areas of solid tumor. Note that total percentage is 106.9% since some tumor cells demonstrated both a cytoplasmic and basal staining pattern. (**D**) Pie chart depicting the average percentage of PLEKHA7 expression as lost (68.6%) or apical (31.4%) in the regions of glandular tumor from all IBC patient samples. No cytoplasmic or basal staining of PLEKHA7 was observed in areas of glandular tumor. (**E**) Expression of PLEKHA7 by immunohistochemistry (IHC) in normal breast tissue. Scale bar = 100 µm in all images. (**F**–**I**) Examples of expression patterns for PLEKHA7 in IBC patient samples. (**F**) Apical staining, (**G**) loss of staining, (**H**) cytoplasmic staining, and (**I**) basal staining. (**J**) A representative gel image demonstrating expression of PLEKHA7, E-cadherin, and p120-catenin by Western blot is shown.

To start assessing the role of PLEKHA7 in IBC, we determined its expression pattern by immunohistochemistry (IHC) in IBC patient samples. Archival surgical pathology material from 62 patients with a diagnosis of IBC was recovered from the Institutional Tissue Registry from Mayo Clinic Minnesota, Florida, and Arizona campuses and evaluated for adequacy. Sixteen samples were excluded from analysis either due to lack of appropriate tissue (e.g., small representation of neoplastic population) or due to IHC technical issues (e.g., loss of neoplastic population on IHC slides or failure of IHC staining, as shown by lack of staining in normal ducts that served as our internal control). Interpretation of hematoxylin and eosin (H & E) and IHC slides was performed by an independent pathologist. Two main morphological patterns were observed: solid and glandular. The predominant pattern of growth was solid, with sparse glandular formations (see Figure S1 for examples). Five tumors demonstrated only the solid pattern of growth with no glandular formations. Tumors were divided into predominantly solid (75–100% of tumor exhibits solid pattern of growth), solid (25–74% of tumor exhibits solid pattern of growth), and sparsely solid (0–24% of tumor exhibits solid pattern of growth). The distribution of IBC samples based on the predominance of the solid pattern of growth is displayed in Figure 1A. Tumors were also categorized based on the percentage of the glandular pattern of growth into five categories: 0%, 1–5%, 6–25%, 26–50%, and 51–100%.The distribution of IBC samples based on the glandular pattern of growth is displayed in Figure 1B. 

PLEKHA7 expression was distinct between the solid and glandular patterns. For each morphological pattern, the average PLEKHA7 staining pattern across all tumor samples was calculated and categorized based on localization (apical, lost, cytoplasmic, or basal). In solid areas, PLEKHA7 was lost in 56.4%, cytoplasmic in 26.4%, and localized to the basal membrane in 24.1% across all IBC tumor samples (See Figure 1C). Apical staining was not observed in solid areas of the tumor. In contrast, PLEKHA7 staining in glandular areas was either lost (68.6%) or apical (31.4%) (See Figure 1D). Examples of PLEKHA7 staining for each location are shown in Figure 1E–I. See Table S1 for a complete breakdown and analysis of PLEKHA7 expression in IBC samples. It is notable that PLEKHA7 must properly localize to the apical AJs to maintain its tumor suppressing function [19]. Therefore, we anticipated that PLEKHA7 would not be functional in the overwhelming majority of these patient tumors.

To further interrogate the function of PLEKHA7 in IBC, we utilized two frequently used cell line models: SUM149 and SUM190. SUM149 cells belong to the triple negative basal molecular subtype, while SUM190 to the hormone receptor negative, erbB2/Her2 positive molecular subtype. Western blot experiments indicated that both SUM149 and SUM190 cell lines express very low levels of PLEKHA7 protein compared to Caco2 cells, a classic human epithelial cell model for studying the AJs (see Figure 1J). We also tested MCF12A, a non-tumorigenic breast line, for PLEKHA7 expression and localization. We found that this line was not optimal for studying the apical AJ, as under normal culture conditions PLEKHA7 largely localizes to the cytoplasm (see Figure S2B) and is not robustly expressed (see Figure S2C). However, from immunohistochemistry data, we found that PLEKHA7 is expressed and localizes exclusively to apical AJs in breast epithelial cells (see Figure S2A and Figure 1E). Thus, we used Caco2 as a comparison for normal apical AJs. Collectively, these data suggest a consistent loss of functional PLEKHA7 in IBC, despite normal to high expression of p120 and E-cadherin (Figure 1J and previous studies) [5,17]. 

**Figure 2:**
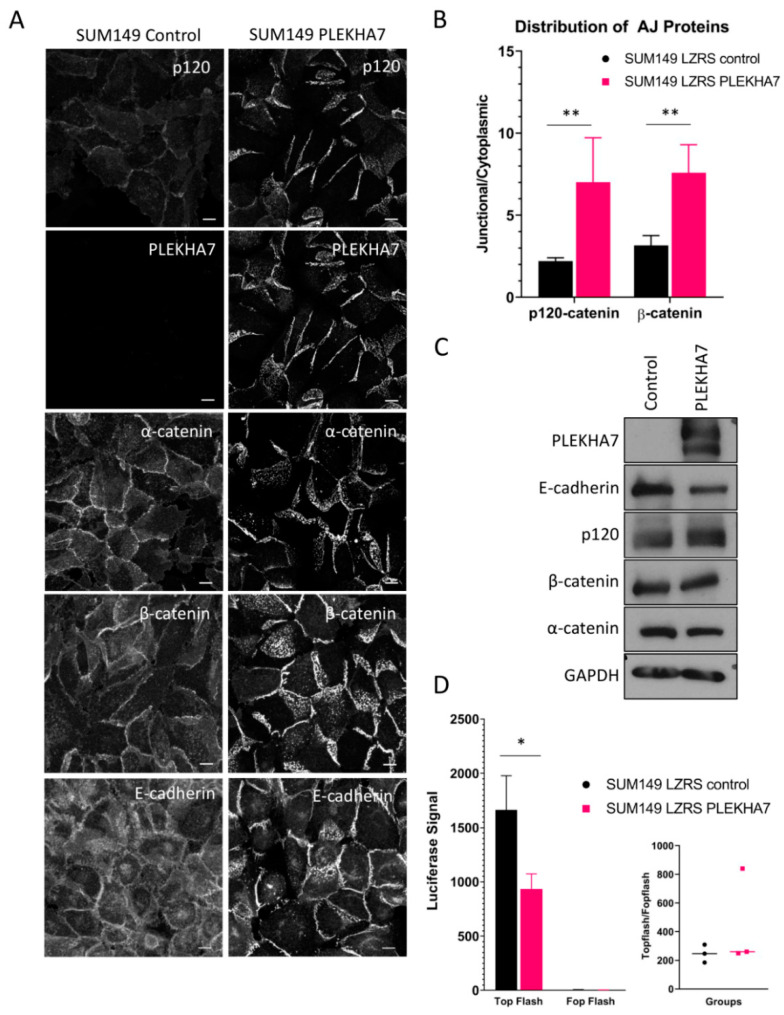
Effect of PLEKHA7 re-expression on SUM149 cell adherens junctions. (**A**) SUM149 cells expressing LZRS ms neo (control) or LZRS PLEKHA7 were grown to confluence and immunofluorescence was performed for E-cadherin, p120-catenin, PLEKHA7, α-catenin, or β-catenin. Images were obtained by confocal microscopy. Images are maximum projection intensity. Scale bar is 10μM. Representative images are shown. (**B**) Intensity of p120-catenin or β-catenin staining is expressed as a ratio of the signal at apical AJs to cytoplasmic staining. N > 75 cells per condition per group. ** indicates *p* < 0.001, Student’s *t*-test (*p* < 0.0001 for both p120-catenin and β-catenin). (**C**) Protein levels of PLEKHA7, E-cadherin, p120-catenin, β-catenin, and α-catenin in SUM149 LZRS ms neo or SUM149 LZRS PLEKHA7 cells were obtained by Western blot. A representative image is shown. No consistent change was observed for any junctional protein in repeated experiments. (**D**) A representative graph displaying Wnt/β-catenin signaling in SUM149 LZRS ms neo and SUM149 LZRS PLEKHA7 cell lines using dual luciferase reporter assay. * indicates *p* < 0.05, Student’s *t*-test (*p* = 0.022).

Next, we used viral transduction to examine the effects of PLEKHA7 re-expression in SUM149 cells. We found that exogenously expressed PLEKHA7 localizes to and strengthens the AJs, as evidenced by increased junctional accumulation of p120, E-cadherin, α-catenin, and β-catenin (see Figure 2A,B). This junctional strengthening is similar to previous reports [19,20,23]. Notably, PLEKHA7 re-expression altered the location but the overall levels of junctional proteins were not consistently changed (see Figure 2C), which is similar to results from previous publications [19,20]. In agreement, we observed decreased cytoplasmic localization of p120 and β-catenin in PLEKHA7-expressing cells (see Figure 2B).

As increased cytoplasmic β-catenin could lead to increased nuclear signaling, we tested for altered activity in the Wnt/β-catenin pathway using the dual luciferase reporter assay (Figure 2D). Although TopFlash activity was reduced in PLEKHA7-SUM149 compared to control, we did not see consistent changes in activity when normalized by the FopFlash reporter (Figure 2D). Therefore, while PLEKHA7 expression increases the junctional localization of β-catenin, it does not affect Wnt/β-catenin nuclear signaling under these conditions in SUM149 cells.

**Figure 3:**
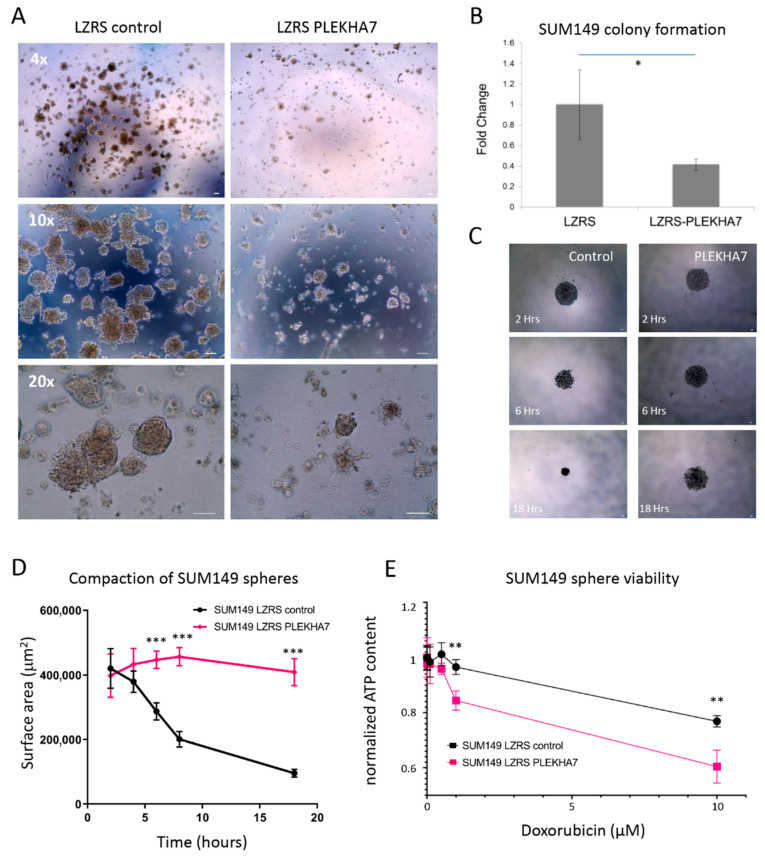
Effects of PLEKHA7 re-expression on SUM149 cell growth and survival in 3D culture. (**A**) SUM149 LZRS ms neo (control) and SUM149 LZRS PLEKHA7 cells were grown in Matrigel for approximately two weeks. Representative images at 4×, 10×, and 20× magnification are shown. Scale bar in each image = 100µm. (**B**) Quantification of SUM149 LZRS ms neo or SUM149 LZRS PLEKHA7 colonies from 3A are shown as the fold change from SUM149 LZRS ms neo. * indicates *p* < 0.05, Student’s *t*-test. (**C**) Representative images from SUM149 LZRS ms neo and SUM149 LZRS PLEKHA7 suspension cultures undergoing sphere compaction over 18 h. Images taken at 4×. Scale bar in each image = 100µm. (**D**) A representative graph of SUM149 LZRS ms neo and SUM149 LZRS PLEKHA7 suspension cultures compacting into spheres over 18 h under ultralow attachment conditions. *** indicates *p* < 0.001, Student’s *t*-test (*p* < 0.0001). (**E**) A representative graph of normalized ATP content produced by SUM149 LZRS ms neo and SUM149 PLEKHA7 spheres after treatment with various concentrations of DOXIL/doxorubicin for 72 h. ** indicates *p* < 0.01, Student’s *t*-test (*p* = 0.002 for 1 µM doxorubicin and 10 µM doxorubicin). Each group was normalized to no treatment.

To test the hypothesis that PLEKHA7 loss in IBC promotes a more aggressive phenotype, we next examined whether restoring PLEKHA7 to the apical AJs suppresses cell growth. Using the MTT assay under 2D culture conditions, PLEKHA7-expressing SUM149 cells exhibited fewer cell numbers compared to SUM149 cells infected with control virus (Figure S3). Further, when PLEKHA7-expressing SUM149 cells were plated on Matrigel, they formed fewer and smaller colonies compared to control SUM149 cells (see Figure 3A,B). IBC patients frequently demonstrate tumor emboli in the dermal lymphatics, and spheroid formation under ultralow attachment conditions has been used as a model of IBC tumor emboli [7]. SUM149 cells infected with control virus rapidly formed compact spheres when grown in suspension. In contrast, PLEKHA7-expressing SUM149 were more loosely connected and less compacted than control SUM149 cells (see Figure 3C,D). Interestingly, the ability of IBC cells to form compact spheroids has been correlated directly to their tumorigenic potential [7]. A prior study comparing 2D and 3D drug responses in a variety of non-IBC breast cancer cell lines found increased chemoresistance in several 3D cultures compared to 2D ones. The authors correlated the chemoresistance with the ability of cell lines to form dense, compact spheres in 3D [24]. Thus, we hypothesized that the less compacted spheres would be more vulnerable to chemotherapy treatment. To test this, we determined the sensitivity of control and PLEKHA7-expressing SUM149 spheres to DOXIL, the liposomal formulation of doxorubicin hydrochloride, one of the standard, first-line neoadjuvant chemotherapy agents used in IBC treatment [3]. A regression model analysis was performed for differences in ATP content between control and PLEKHA7-expressing SUM149 spheres. Notably, after 72 h of treatment, PLEKHA7-SUM149 spheres were significantly less viable than control SUM149 spheres in response to doxorubicin treatment (see Figure 3E).

**Figure 4:**
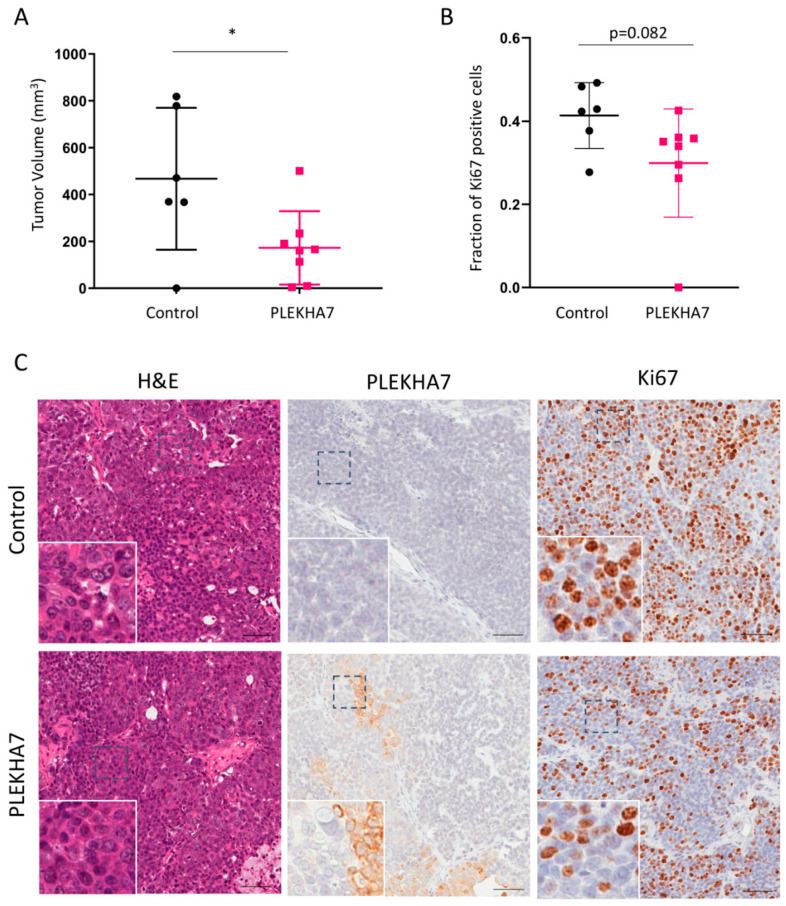
PLEKHA7 effects on SUM149 tumor growth in orthotopic xenografts. (**A**) Tumor volume of xenografts from SUM149 LZRS ms neo (control) or SUM149 LZRS PLEKHA7 cells implanted into the fourth mammary gland of NOD/SCID immunocompromised mice measured at eight weeks post-implantation. * indicates *p* = 0.034, Student’s *t*-test. *n* = 6 for control group, *n*= 8 for PLEKHA7 group. (**B**) Fraction of Ki-67 positive cells in tumors from SUM149 LZRS ms neo or SUM149 LZRS PLEKHA7 xenografts at the eight week endpoint. *p* = 0.082, Student’s *t*-test. *n* = 6 for control group, *n*= 8 for PLEKHA7 group. (**C**) Representative IHC images from SUM149 LZRS ms neo or SUM149 LZRS PLEKHA7 xenografts for H & E, PLEKHA7, and Ki67. Scale bar represents 100 µm.

Our in vitro and IHC data indicated that PLEKHA7 acts as a tumor suppressor and is frequently misregulated in IBC. Next, we tested whether restoring PLEKHA7 expression in SUM149 cells would decrease tumor formation or growth in an animal model. SUM149 cells reliably form tumors in xenograft models when injected orthotopically [17,25]. PLEKHA7-expressing SUM149 cells or control SUM149 cells were injected into the fourth mammary fat pad of NOD/SCID immunocompromised mice, and mice were monitored for eight weeks for the presence and size of tumors formed. Mouse body weight changes were not observed in either group. After eight weeks, mice were sacrificed and tumors were obtained for IHC. As shown in Figure 4A,B, tumors in the PLEKHA7-expressing SUM149 mice were smaller and less proliferative than control. Importantly, IHC analysis revealed that PLEKHA7 expression was commonly misregulated in the PLEKHA7-expressing tumors. After eight weeks, we found that most of the PLEKHA7-tumors had lost significant expression of PLEKHA7, retaining between 15–55% PLEKHA7 depending on the mouse. Furthermore, we frequently observed cytoplasmic PLEKHA7 staining, with only approximately 10–25% junctional PLEKHA7 remaining in most tumors. An example is shown in Figure 4C. This indicates a negative selection of tumor cells expressing junctional PLEKHA7. Accordingly, when we quantified tumors for changes in Snail, Myc, and Cyclin D1, proteins that have been previously shown to be suppressed by PLEKHA7 function in Caco2 cells [19], we observed a trend towards reduced expression in PLEKHA7-SUM149 tumors that did not reach significance (see Figure S4A–C). We hypothesize that the tumor suppressive effects observed with PLEKHA7 expression in SUM149 xenografts occurred early in tumor formation. This early effect hampered tumor growth sufficiently enough to enable overall differences in tumors between groups to be observed. However, PLEKHA7-positive tumors escaped these suppressive effects by deregulating ectopically expressed PLEKHA7 throughout the eight week course of the experiment. 

## 3. Discussion

Understanding the molecular alterations contributing to the uniquely aggressive phenotype and clinical course of IBC has proven quite challenging. E-cadherin and p120-catenin are required for IBC tumor growth and emboli formation but it is not known whether the apical or basolateral cadherin–catenin complexes drive IBC. In this study, we began to examine the functionality of the apical cadherin–catenin complex. Specifically, we tested and demonstrated that restoration of apical complex protein PLEKHA7 strengthens the apical AJ and inhibits the aggressive nature of IBC. 

E-cadherin is considered a tumor suppressor and loss of E-cadherin (*CDH1*) expression is a cardinal feature of invasive lobular carcinoma of the breast [26,27]. In mouse models, E-cadherin loss causes invasive lobular carcinoma due to anoikis resistance, increased angiogenesis and increases in growth factor signaling [28,29].

Nonetheless, strong expression of E-cadherin is consistently observed in IBC primary tumors and tumor emboli in the dermal lymphatics [5]. Evidence in animal and in vitro models suggests a causative role for E-cadherin in maintaining cohesion of tumor emboli and tumor growth [7,17]. Silvera et al. also identified a tumor-promoting and emboli-forming role for p120 in IBC cell line and xenograft models [17], which was attributed to its ability to promote the stability and junctional retention of E-cadherin. 

There is also increasing evidence that E-cadherin complexes can promote tumor growth and metastasis in non-IBC invasive ductal carcinomas (IDC) [30,31]. Using xenograft models from a triple negative IDC cell line, E-cadherin expression favored primary tumor growth, while E-cadherin knockdown had the opposite effect [32]. An extensive study exploring the effect of E-cadherin on metastasis across multiple animal models of breast cancer demonstrated that E-cadherin is necessary for metastasis in luminal and basal models of IDC [30].

These findings highlight the role of collective cell migration in the metastatic process, and the function of adhesion complexes in maintaining intercellular contacts during this process. Mammosphere formation across various breast cancer lines demonstrated a positive correlation with E-cadherin expression [33]. Consistently, E-cadherin function-blocking antibodies inhibited the size and number of tumor emboli in the vessels of an IBC mouse model [7].

Recent work from our lab determined distinct functional roles for apical vs. basolateral cadherin–catenin complexes. Basolateral cadherin–catenin complexes promote AIG and expression of pro-tumorigenic factors. Apical cadherin–catenin complexes suppress expression of these factors and inhibit AIG through a mechanism that depends on PLEKHA7 [19]. We postulated that the aggressive nature of IBC is mediated by an imbalance between the tumor promoting basolateral and the tumor suppressing apical cadherin–catenin complexes. We tested this hypothesis by focusing on the expression and function of PLEKHA7 in IBC. We found that PLEKHA7 expression is largely not apical in IBC tumors, indicating a loss of the tumor-suppressing function in this disease. 

This is highly consistent with previous reports that PLEKHA7 is lost or mislocalized in invasive ductal carcinoma [19,34,35]. We and others have found that PLEKHA7 is primarily cytoplasmic in invasive lobular carcinoma, presumably due to E-cadherin loss [34], while in invasive ductal carcinoma, PLEKHA7 expression is predominantly lost. To reiterate, the loss or mislocalization of PLEKHA7 from the apical AJ in the context of functional basolateral cadherin–catenin complexes is expected to promote tumor properties [19]. In IBC, we have observed the selective disruption of PLEKHA7 in patient tumor samples, with PLEKHA7 loss being twice as common as cytoplasmic mislocalization. While deregulation of PLEKHA7 is not specific to inflammatory breast cancer, IBCs consistently express and require E-cadherin and p120 for their growth and aggressive behavior. Our previous characterization of E-cadherin and p120 across non-IBC breast cancers of varying types and stages showed quite variable expression and localization of E-cadherin and p120 [19]. 

Both IBC cell lines tested revealed low PLEKHA7 expression. Ectopic expression of PLEKHA7 was largely junctional and resulted in strong recruitment of cadherin–catenin complex proteins to PLEKHA7-positive junctions, in agreement with previous studies [19,20,23]. Interestingly, PLEKHA7 re-expression redistributed p120 and β-catenin away from the cytoplasm and into the apical AJ. While not tested directly here, this may have functional consequences as cytoplasmic localization of p120 can suppress RhoA activity (Reviewed in [36]). We did not, however, observe consistent changes in β-catenin nuclear activity between PLEKHA7-expressing or control SUM149 cells. Increased junctional and decreased cytoplasmic β-catenin localization upon PLEKHA7 expression was also observed in an ovarian cancer model but alterations to β-catenin-nuclear activity were not tested in that study [37]. 

Consistent with the hypothesis that PLEKHA7 acts as a tumor suppressor that is commonly lost in IBC, its re-expression in an IBC cell line significantly inhibited cell growth, both in 2D and particularly in 3D culture. Our data also indicate that PLEKHA7 re-expression suppresses both the number and size of SUM149 colonies on Matrigel. We did not test the mechanism by which PLEKHA7 executes these tumor-suppressor roles in IBC. Previous studies determined that PLEKHA7 recruits the RNAi machinery to the apical AJs to promote the maturation of miRNAs and their association with an apically localized RISC [19,22]. This function of PLEKHA7 suppressed expression of pro-tumor promoting proteins, including Cyclin D1, Snail, and c-Myc, and inhibited AIG [19]. Recent work in colon cancer patient samples demonstrated concomitant dysfunction of PLEKHA7 and the RNAi machinery at the apical AJs during colon cancer progression [38]. When PLEKHA7 was restored in colon cancer cell lines, the RNAi machinery was also restored to apical AJ and PLEKHA7-expressing xenografts showed a reduced tumor burden [38]. 

We cannot exclude the possibility that other mechanisms contribute to the tumor suppressive function of PLEKHA7 in IBC, including inhibiting p120 signaling, or suppressing E-cadherin/Epidermal growth factor receptor (EGFR) signaling [37]. In certain ovarian epithelial cancer lines, E-cadherin has been shown to promote EGFR signaling, and this is suppressed by expression of PLEKHA7 [37]. The ability of E-cadherin to promote EGFR signaling in IBC is unexplored. Approximately 30% of IBC patients exhibit EGFR-positive tumors, which correlates with worse overall survival [39]. If E-cadherin potentiates EGFR signaling in IBC, re-expression of PLEKHA7 may suppress this signaling axis. 

Re-expression of PLEKHA7 suppressed in vitro spheroid compaction in 3D culture. Increased cohesion of tumor emboli is a key characteristic of IBC. We observed that suppression of sphere compaction in PLEKHA7-expressing cells correlated with decreased survival after treatment with doxorubicin, a standard neoadjuvant chemotherapy used in IBC treatment. This finding was consistent with a previous study that correlated spheroid compaction with resistance to drug treatment [24]. A recent study discovered the presence of “nanolumina” between clustered breast cancer cells [40]. These nanolumina are very small, impermeable intercellular spaces bound by cell junctions in compacted tumor cell clusters. Nanolumina promote high local concentration of pro-growth signals, which contributed to increased metastasis by tumor cells organized in clusters [40]. It is possible that nanolumina compartments between cells within compact spheres promote signaling that renders the tight clusters more resistant to chemotherapy than the more loosely connected spheres. Another non-mutually exclusive possibility is that PLEKHA7 alters the number of cancer stem cell (CSC)-like cells in the SUM149 spheres. It is well-described that cancer spheres contain CSC-like cells and this population of cells has been linked to chemoresistance. We previously reported that one of the top pathways PLEKHA7 may regulate by RNAi is human embryonic stem cell pluripotency [22]. Therefore, it is possible that PLEKHA7 expression reduces the number of CSC-like cells, rendering SUM149-PLEKHA7 spheres less viable to doxorubicin treatment. 

In orthotopic xenograft models, re-expression of PLEKHA7 slowed tumor growth, leading to decreased tumor volume. Notably, by the end of our eight week study, we also observed that the majority of PLEKHA7-expressing tumors had bypassed PLEKHA7 tumor-suppression by losing PLEKHA7 or causing a cytoplasmic localization. This suggests the existence of selective pressure in tumor cells to lose apical PLEKHA7 expression.

Altogether, our data is consistent with an important tumor suppressor function for PLEKHA7 in IBC. While the mechanisms of its loss or mislocalization away from the apical AJs are still unclear, PLEKHA7 dysfunction is almost universal in IBC patient samples. Re-expression of PLEKHA7 restores apical AJs and suppresses tumor growth in vitro and in vivo. These data suggest a more nuanced understanding of the role that the cadherin–catenin complex plays in promoting IBC. Specifically, our data indicate that IBC growth and emboli formation are promoted by the abundance of basolateral cadherin–catenin complexes and the lack of tumor-suppressive apical cadherin–catenin complexes. 

## 4. Materials and Methods

### 4.1. Constructs

The full-length human PLEKHA7 construct utilized in this study was previously described [19]. The LZRS ms neo plasmid was a generous gift from Dr. Albert Reynolds (Vanderbilt University, Nashville, TN, USA). TopFlash and FopFlash reporter plasmids were a kind gift from Dr. Aubrey Thompson (Mayo Clinic Florida) and were originally acquired from Upstate Cell Signaling Solutions ((21–170) and (21–169)). Renilla-TK plasmid (pGL4.74, hRluc/TK) was purchased from Promega (E6921).

### 4.2. Immunofluorescence

Cells were grown to confluence on glass coverslips and fixed by either 100% methanol or 4% paraformaldehyde/4% sucrose. Coverslips were rinsed with PBS and cells fixed with methanol in −20 °C for 7 min. Alternatively, coverslips were rinsed with PBS, fixed in paraformaldehyde at room temperature for 15 min, washed twice with PBS/10 mM glycine for 5 min each, and solubilized with PBS containing 0.2% Triton X-100 for 5 min. Coverslips were blocked with Dako protein block (Agilent, Santa Clara, CA, USA) and incubated with primary antibody diluted in Dako antibody diluent (Agilent) overnight. Coverslips were washed three times in PBS, exposed to secondary antibodies for 1 h, washed once in PBS, washed in PBS + 4, 6-Diamidino-2-phenylindole dihydrochloride (DAPI) (Sigma Aldrich, St. Louis, MO, USA, D8417) to stain nuclei, followed by two additional PBS washes. Coverslips were then mounted on glass slides with Aqua Poly/Mount (Polysciences, Warrington, PA, USA) and imaged using a 63× oil objective on a Carl Zeiss LSM 800 confocal microscope. Z-stacks were taken using 0.5 μm intervals. All image processing, including generation of maximum projection intensity images and addition of scale bars, was completed with Zen Black or Zen Blue software (Carl Zeiss Microscopy, Oberkochen, Germany). Primary antibodies included: anti-PLEKHA7 (Sigma Aldrich, HPA038610), anti-p120-catenin (15D2, a kind gift from Dr. Albert Reynolds), anti-α-catenin (Abcam, Cambridge, UK, ab231306), anti-β-catenin (BD Transduction Laboratories, San Jose, CA, USA, 610154), and anti-E-cadherin (BD Transduction Laboratories, 610182). Secondary antibodies included goat anti-rabbit IgG Alexa 488 (Thermo Fisher Scientific, Waltham, MA, USA, A-11034), goat anti-mouse IgG Alexa 488 (Thermo Fisher Scientific, Waltham, MA, USA, A-11029), goat anti-rabbit IgG Alexa 594 (Thermo Fisher Scientific, Waltham, MA, USA A-11037), and goat anti-mouse IgG Alexa 594 (Thermo Fisher Scientific, Waltham, MA, USA A-11032).

Analysis of junctional and cytoplasmic staining of p120-catenin and β-catenin staining was performed using Fiji (Fiji is Just ImageJ, [23]) on maximum projection intensity images. Regions of interest were drawn to contain areas of cytoplasmic staining and exclude areas of apical or basolateral cadherin–catenin staining. Mean pixel intensity was calculated. Linear regions of interest were drawn for apical AJ staining and mean pixel intensity was calculated. Background pixel intensity measurements were subtracted from each. All cytoplasmic staining was averaged for all the cells in a single image and divided by the average junctional intensity for the cells in the same image. Mean apical junctional staining/cytoplasmic staining was calculated for at least 75 cells per group (a total of 9–11 images) for p120-catenin and β-catenin. 

### 4.3. Western Blot

Cells were plated to confluence (Caco2, SUM149) or high density (SUM190, according to manufacturer). Before lysis, cells were rinsed with PBS and lysed with one of the following buffers: RIPA buffer (150 mM NaCl, 50 mM Tris pH 7.4, 0.5% deoxycholic acid, 0.1% SDS, 1% NP-40), Triton-X 100-based buffer (150 mM NaCl, 2 mM EDTA, 25 mM Tris pH 7.4, 0.5% Triton-X 100), or 2× Laemmli sample buffer. For RIPA and Triton-X 100 buffers, 1× protease (Halt’s protease inhibitor cocktail, Thermo Fisher Scientific, Waltham, MA, USA) and phosphatase (Halt phosphatase inhibitor cocktail, Thermo Fisher Scientific, Waltham, MA, USA) inhibitors were added. Lysis with RIPA and Triton-X 100-based buffers was completed on ice. Cells were scraped and lysates passed through a blunt end needle and centrifuged at 4 °C. Protein was quantified using the Pierce BCA Protein Assay (Thermo Fisher Scientific, Waltham, MA, USA). For lysis with 2× Laemmli sample buffer, cells were scraped and lysates passed through a 25G needle. Protein was quantified using the RC DC Protein Assay (Bio-rad, Hercules, CA, USA). Before loading, lysates generated from RIPA or Triton-X 100-based buffers were brought to a final concentration of 2× Laemmli sample buffer and boiled for 5 min. Samples were separated using 8 or 12% SDS-PAGE gels and transferred to nitrocellulose membranes. Membranes were blocked with 3–5% milk/TBST or 3–5% BSA/TBST and incubated overnight with primary antibodies. Membranes were washed three times with TBST, incubated for one hour with secondary antibody, washed three times with TBST and incubated with ECL (GE Healthcare). Membranes were imaged onto autoradiography film (Genesee). 

Primary antibodies included: anti-PLEKHA7 (Sigma Alrich, HPA038610), anti-GAPDH (Cell Signaling, Danvers, MA, USA 2118), anti-β-actin (Cell Signaling, 4967), anti-p120-catenin (15D2, a kind gift from Dr. Albert Reynolds at Vanderbilt University and Millipore Sigma, 05-1567), anti-α-catenin (Sigma Aldrich, C2081), anti-β-catenin (Sigma Aldrich, C2206), and anti-E-cadherin (Cell Signaling, 3195). Secondary antibodies included donkey anti-rabbit igG-HRP (Jackson ImmunoResearch, West Grove, PA, USA 711-035-152) and donkey anti-mouse IgG-HRP (Jackson ImmunoResearch, West Grove, PA, USA, 715-035-150).

### 4.4. Matrigel

Eight-chamber slides (LabTek II) were coated with growth factor-reduced Matrigel (BD) and allowed to solidify. A total of 2500 cells per well were suspended in 2% growth-factor reduced Matrigel and plated. Colonies/spheres formed for approximately two weeks, with regular media changes every two to three days. Images were taken using an AMG EVOS digital inverted microscope and the number of spheres was counted for each well. 

### 4.5. Sphere Compaction

2500 cells per well were plated in 96-well round bottom ultra-low attachment plates (Corning, Corning, NY, USA). Images were taken at 4× using an AMG EVOS digital inverted microscope at the indicated time points. Images were analyzed in Fiji [23] after conversion to 8-bit images. The threshold was adjusted to include only cells and the area was measured using the measurement tool in Fiji. A representative sample was analyzed and presented. Average area was calculated for 15–18 spheres per group, depending on the time point. 

### 4.6. Cell Lines

The Caco2 and MCF12A cell lines were acquired from ATCC. SUM149 and SUM190 cell lines were acquired from Asterand Bioscience (now BIOIVT). All cell lines tested negative for mycoplasma contamination (MycoAlert Mycoplasm detection kit, Lonza AMAXA). Caco2 cells were grown in MEM Eagle with Earle’s salts and L-glutamine (Corning) with 10% fetal bovine serum (Gibco, Waltham, MA, USA), 1× MEM nonessential amino acids (Corning), and 1 nM sodium pyruvate (Gibco). MCF12A cells were grown in DME/F12 (1:1) (Hyclone/Cytiva, Marlborough, MA, USA) with 5% horse serum (Invitrogen), 20 ng/mL human epidermal growth factor (Peprotech, Rocky Hill, NJ, USA), 100 ng/mL cholera toxin (Sigma Aldrich), 0.01 mg/mL insulin (Sigma Aldrich), and 500 ng/mL hydrocortisone (Sigma Aldrich). SUM149 and SUM190 cells were grown according to BioIVT instructions. Specifically, SUM149 cells were grown in Ham’s F-12 (Gibco) supplemented with 5% heat-inactivated fetal bovine serum (Gibco), 10 mM HEPES (Lonza), 1 µg/mL hydrocortisone (Sigma Aldrich), and 5 µg/mL insulin (Sigma Aldrich). SUM190 cells were grown in Ham’s-F-12 (Gibco) supplemented with 1 g/L bovine serum albumin (Sigma Aldrich), 5 mM ethanolamine (Sigma Aldrich), 10 mM HEPES (Lonza), 1 µg/mL hydrocortisone (Sigma Aldrich), 5 µg/mL insulin (Sigma Aldrich), 8.7 ng/mL sodium selenite (Sigma Aldrich), 5 µg/mL apo-transferrin (Sigma Aldrich), and 6.7 ng/mL triiodo-L-thyronine (T3) (Sigma Aldrich). At time of culturing, a final concentration of 2% heat-inactivated fetal bovine serum (Gibco) was added to media for culturing SUM190 cells.

### 4.7. MTT Assay

2500 cells/well were plated in flat-bottom 96-well microplates (Fisher). At 24 h, and every 24 h thereafter, the MTT (Thiazolyl Blue Tetrazolium Bromide) cell viability assay was performed as follows: fresh media was added to cells followed by addition of 0.83 mg/mL MTT reagent, dissolved in PBS. Reaction proceeded for exactly one hour and was stopped by addition of DMSO. Absorbance was read at 550 nm using a Flexstation 3 Spectrophotometer. 

### 4.8. Drug Treatment

2500 cells/well were plated in round-bottom ultra-low attachment 96 well microplates (Corning) with Microclime lid (Labcyte, Thermo Fisher Scientific). At 18 h, DOXIL, the liposomal formulation of doxorubicin hydrochloride, was added at indicated concentrations and cells were incubated for an additional 72 h. Cellular ATP was assessed using the Cell Titer Glo assay (Promega). For this assay, an equal volume of Cell Titer Glo Reagent was added to wells containing cell spheres. The plate was shaken at 55 rpm for five minutes and the entire volume was transferred to an opaque plate for reading. Luminescence was read on a Veritas microplate luminometer. ATP content was calculated based on a standard ATP curve. DOXIL was obtained from the Mayo Clinic Pharmacy in Jacksonville, Florida. 

### 4.9. Dual Luciferase Reporter Assay 

SUM149 LZRS ms neo or SUM149 LZRS PLEKHA7 cells were plated to 40–50% confluence and transfected with TopFlash and Renilla-TK, or FopFlash and Renilla-TK reporter plasmids using jetPRIME DNA transfection reagent (Genesee Scientific), according to the manufacturer’s instructions. Cells were lysed using the passive lysis buffer provided in the Dual-Luciferase Reporter Assay system (Promega) and the dual luciferase reporter assay was performed according to manufacturer’s instructions. Luciferase signal was read on a Veritas microplate luminometer. TopFlash and FopFlash luciferase signals were normalized to the Renilla-TK signal. Further analysis was performed by normalizing the TopFlash signal to the FopFlash signal.

### 4.10. Animal Experiments

All animal experiments were reviewed and approved by the Institutional Animal Care and Use Committee (IACUC) at Mayo Clinic on 23 May 2018. All experiments and procedures adhered to the guidelines approved by IACUC (Protocol: A00003637-18). NOD/SCID mice aged eight- to ten-weeks-old were obtained from Jackson Laboratories. SUM149 cells tested negative for Ectromelia, LCMV, LDEV, MHV, MPV, MVM, Mycoplasma pulmonis, Mycoplasma sp, Polyoma, and TMEV (IDEXX BioResearch). Injection were made into the fourth mammary fat pad with 5 × 10^5^ SUM149 LZRS control (nice mice) or SUM149 LZRS PLEKHA7 cells (eight mice) mixed with Matrigel (BD Transduction Lab) at 1:1. All cells also expressed a luciferase reporter plasmid. Animals were checked daily Monday–Friday for any signs of distress or excessive tumor burden. Tumor volume was measured weekly using a caliper. Luciferase signal was measured weekly using Caliper Life Sciences IVIS imaging system. One control mouse died unexpectedly during the course of the study and was excluded. After eight weeks, mice were sacrificed and the tumor was collected and fixed in formalin. Prior to fixation, tumor volume and weight were measured using caliper and microscale, respectively. Two mice in the control group were excluded due to lack of tumor formation (no tumor was visually observed or palpable and no tumor cells were found by immunohistochemistry of tissue taken from the mammary fat pad). 

### 4.11. Immunohistochemistry—Ethics Statements

All of the IBC patient samples were originally obtained under the 09-001909 and 08-004581 protocols approved by the Mayo Clinic Institutional Review Board. All patients were consented for tissue collection intended for research purposes. Patient tissue samples were de-identified. Utilization of paraffin-embedded tissue samples for the current research study was performed under the 675-05 protocol, also approved by the Mayo Clinic Institutional Review Board. Original date of approval was 8 March 2007. The Mayo Clinic Institutional Review Board deemed the experiments proposed and performed herein as minimal risk and did not require further consent. A summary of cellular morphology and PLEKHA7 expression is included in Table S1.

### 4.12. Immunohistochemistry

Tissue slides were deparaffinized in xylene and rehydrated with ethanol. Antigen retrieval was performed according to the manufacturer protocol (DAKO) with citrate buffer pH 6.0 (PLEKHA7, Ki67, Snail) or EDTA pH 9.0 (Cyclin D1, c-Myc). Slides were incubated with primary antibody for one hour and rinsed with TBST, followed by incubation with secondary antibody for 30 min and additional wash with TBST. To stain nuclei, slides were incubated with 3, 3′-diaminobenzidine (DAB) (DAKO) followed by Gills I hematoxylin. Primary antibodies included PLEKHA7 (Sigma, HPA038610), Cyclin D1 (Cell Signaling, 2978), Snail (Cell Signaling, 3879), c-Myc (Abcam, ab32072), and Ki67 (DAKO, M7240).

Slides were scanned using Aperio ScanScope XT (Leica) and viewed using Aperio eSlideManager 12.4.2.5010 and Aperio ImageScope 64 v12.4.2.7000 (Leica). Images were extracted for analysis using Aperio ImageScope 64 v12.4.2.7000 (Leica). 

PLEKHA7 staining was analyzed by an independent pathologist. Sixteen samples were excluded due to either lack of staining on internal controls or very few tumor cells found on the slide. IBC tumor samples contained solid and/or glandular cellular patterns based on H & E and PLEKHA7 immunohistochemistry. Each tumor sample was characterized based on the percentage of solid or glandular pattern. The percentage of PLEKHA7 expression and localization was then determined for each cellular pattern. PLEKHA7 staining had the following patterns: 1) normal/apical membrane, 2) lost, 3) cytoplasmic, 4) basal. 

For presentation of the data, an average of the percent of PLEKHA7 expression for each of the above four staining patterns was calculated, for either solid or glandular cellular patterns. In the solid areas, the total PLEKHA7 staining added up to 106% due to some tumor cells demonstrating both a cytoplasmic and basal staining. 

To quantify Ki-67, Cyclin D1, Snail, and c-Myc staining of xenograft samples, five to eight representative and randomly dispersed images of 500 × 500 arbitrary units were captured from each sample using Aperio ImageScope 64 v12.4.2.7000 (Leica). Total nuclei and Ki67, Cyclin D1, Snail, or c-Myc positive nuclei were counted in Adobe Photoshop cc 2018 using the counting tool. Ki67, Cyclin D1, Snail, or c-Myc positivity was expressed as a fraction of total nuclei and an average from the images was taken for each sample.

### 4.13. Statistical Methods

All experiments were performed a minimum of three times independently and representative images/experimental data are shown and noted in figure legends. Dual luciferase, Matrigel, and drug treatment assays had between 2–4 biological replicates per experiment. Spheroid compaction assay analysis was performed with a minimum of eight biological replicates per group per time point. Comparisons between control and PLEKHA7 groups were performed using a two-sample t-test. For doxorubicin treatment, a regression model was fit with ATP content as the outcome and group (control/PLEKHA7), doxorubicin dose, and the group by doxorubicin dose interaction as independent variables to determine if the group effect was dependent on dose. Groups were then compared at each dose level using a two-sample t-test. *p*-values <0.05 were considered statistically significant.

## 5. Conclusions

Inflammatory breast cancers are characterized by robust expression of E-cadherin and other members of the cadherin–catenin axis, including p120. These proteins are thought to promote IBC survival and metastasis by maintaining cohesive, viable tumor emboli in the dermal lymphatics. We provide new insights into this characterization by demonstrating that the apical adherens junction complex, characterized by the presence of PLEKHA7, is disrupted in IBC. In the absence of sufficient negative regulation by the apical complex, we anticipated that the abundant basolateral cadherin–catenin complex promotes the aggressive nature of IBC tumors. In support, our data showed that restoring apical complex activity, via PLEKHA7, is sufficient to suppress IBC tumor growth in vitro and in vivo. 

## Data Availability

The data presented in this study are available in Table S1 and contained herein.

## Supplementary Materials

Supplementary materials can be found at https://www.mdpi.com/1422-0067/22/3/1275/s1. Figure S1: Tumor patterns observed in IBC patient samples; Figure S2: PLEKHA7 expression and localization; Figure S3: Effects of PLEKHA7 re-expression in SUM149 cell growth in 2D culture; Figure S4: Expression of Cyclin-D1, Snail, and c-Myc in xenograft tumors; Table S1: Characterization of IBC Patient Samples.

## Abbreviations

AIG	Anchorage independent growth
AJ	Adherens junction
CSC	Cancer stem cell
EGFR	Epidermal growth factor receptor
IACUC	Institutional Animal Care and Use Committee
IBC	Inflammatory breast cancer
IDC	Invasive ductal carcinoma
IHC	Immunohistochemistry
IRES	Internal ribosomal entry site
miRNA	micro-RNA
RISC	RNA-induced silencing complex
RNAi	RNA interference
