# Supplementary material for: PLEKHA7, an Apical Adherens Junction Protein, Suppresses Inflammatory Breast Cancer in the Context of High E-Cadherin and p120-Catenin Expression
Source: Int J Mol Sci. 2021 Jan 28;22(3):1275. doi: 10.3390/ijms22031275 (PMC7865280; doi:10.3390/ijms22031275)
Supplement: Supplementary file 1 [file ijms-22-01275-s001.pdf]

Supplementary

PLEKHA7, An Apical Adherens Junction Protein, Suppresses Inflammatory Breast Cancer in the Context of High E-cadherin and p120-catenin Expression

Lindy J. Pence [1], Antonis Kourtidis [2], Ryan W. Feathers [1], Mary T. Haddad [1], Sotiris Sotiriou [3], Paul A. Decker [4], Aziza Nassar [5], Idris T. Ocal [6], Sejal S. Shah [7] and Panos Z. Anastasiadis [1,*]

[1] Department of Cancer Biology, Mayo Clinic, Jacksonville, FL 32224, USA; emails: pence.lindy@mayo.edu (L.J.P.); feathers.ryan@mayo.edu (R.W.F.); haddad.mary@mayo.edu (M.T.H.); panos@mayo.edu (P.Z.A.)

[2] Department of Regenerative Medicine and Cell Biology, Medical University of South Carolina, Charleston, SC 29425, USA; kourtidi@musc.edu

[3] Department of Laboratory Medicine and Pathology, Mayo Clinic, Rochester, MN 55901, USA; sotiriou.sotiris@mayo.edu

[4] Department of Health Sciences Research, Mayo Clinic, Rochester, MN 55901, USA; decker.paul@mayo.edu

[5] Department of Laboratory Medicine and Pathology, Mayo Clinic, Jacksonville, FL 32224, USA; nassar.aziza@mayo.edu

[6] Department of Laboratory Medicine and Pathology, Mayo Clinic, Phoenix, AZ 85054, USA; ocal.toljay@mayo.edu

[7] Department of Pathology, Kaiser Permanente, Irvine, CA 92618, USA; sejal.s.shah@kp.org

* Correspondence: panos@mayo.edu; Tel.: 904-953-2609

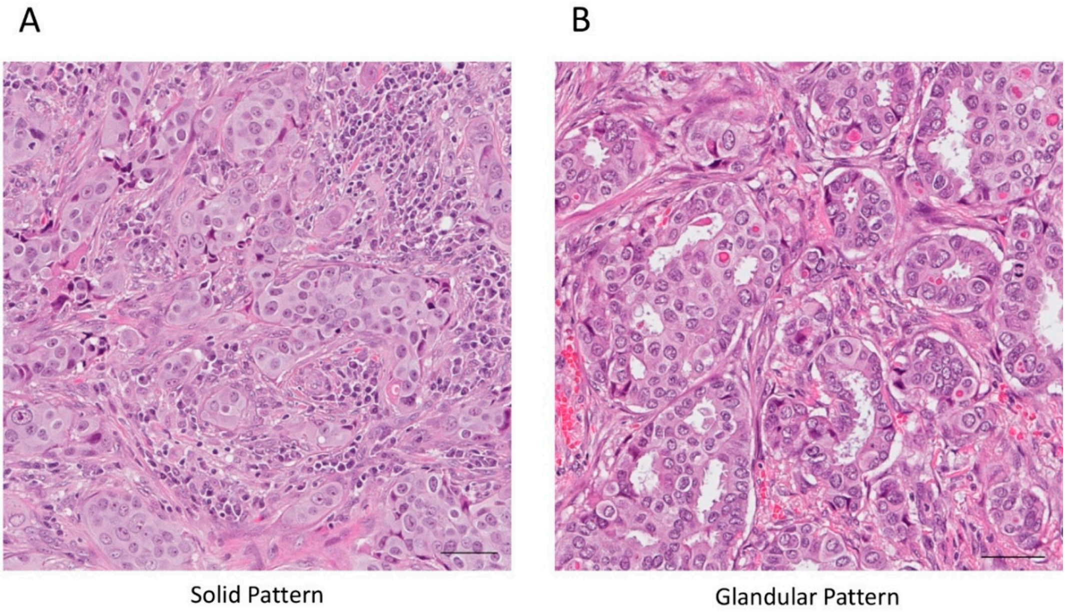

Figure S1. Tumor patterns observed in IBC patient samples. **A**) Example of H&E from IBC tumor demonstrating solid cellular pattern. **B**) Example of H&E from IBC tumor demonstrating glandular cellular pattern. Scale bar represents 100µm.

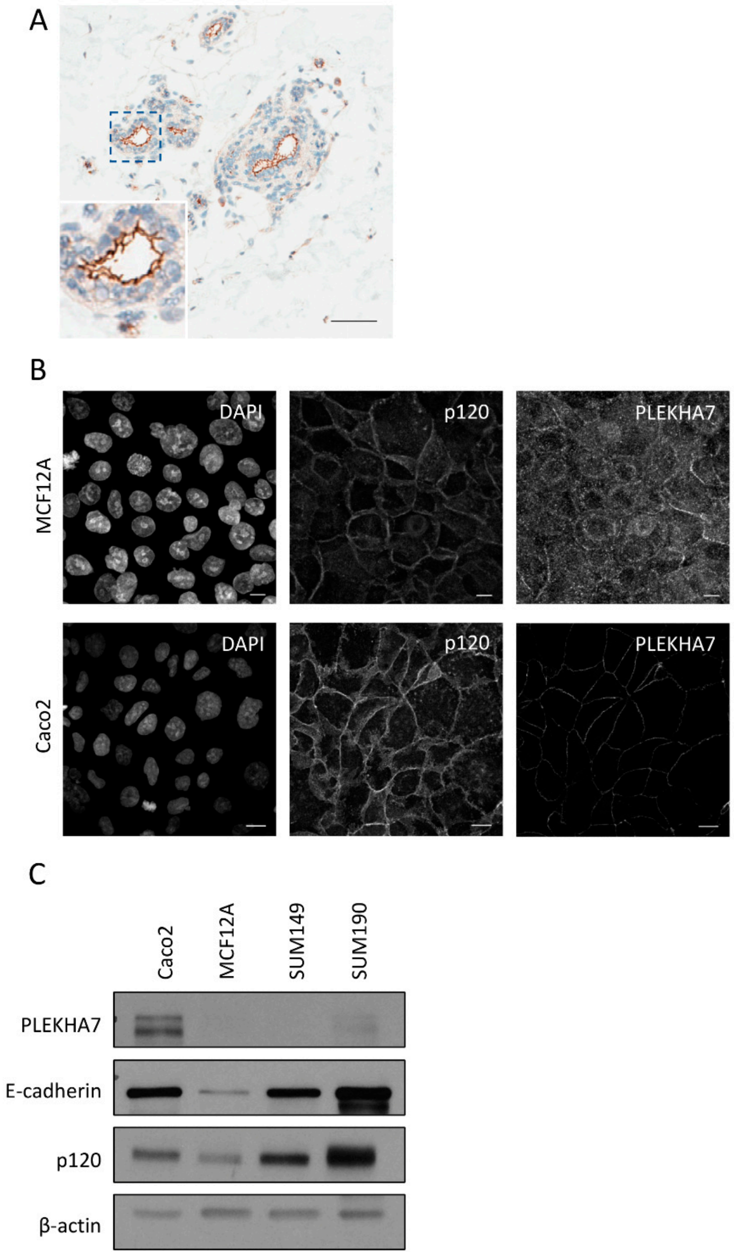

Figure S2. PLEKHA7 expression and localization. **A**) Example of PLEKHA7 staining in a normal breast sample by immunohistochemistry. Scale bar is 100µm. **B**) Immunofluorescence staining of PLEKHA7, p120-catenin and DAPI in MCF12A cell line (scale bar = 10µm) and in Caco2 cell line (scale bar = 20µm). Note: Comparison of intensity is not possible as laser power was different across the two cell lines. **C**). Protein levels of PLEKHA7, E-cadherin, p120-catenin across Caco2, MCF12A, SUM149, and SUM190 cell lines by Western blot. β-actin is used as the loading control.

A

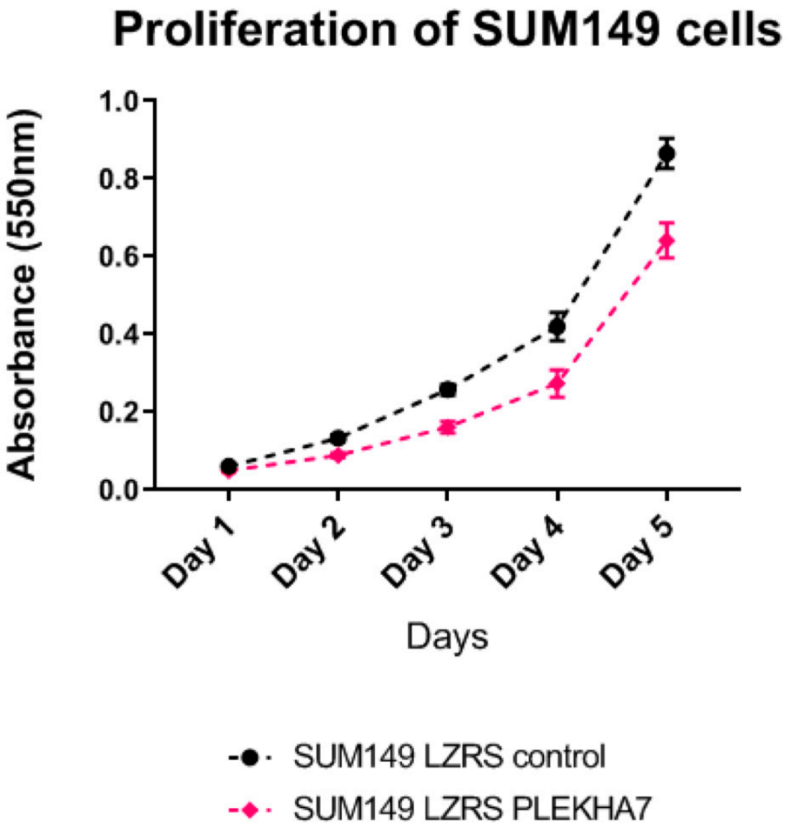

Figure S3: Effects of PLEKHA7 re-expression in SUM149 cell growth in 2D culture. **A**) A representative graph displaying cell growth over time, as measured by the MTT cell metabolic assay, between SUM149 LZRS ms neo (control) and SUM149 LZRS PLEKHA7 cells cultured in 2D.

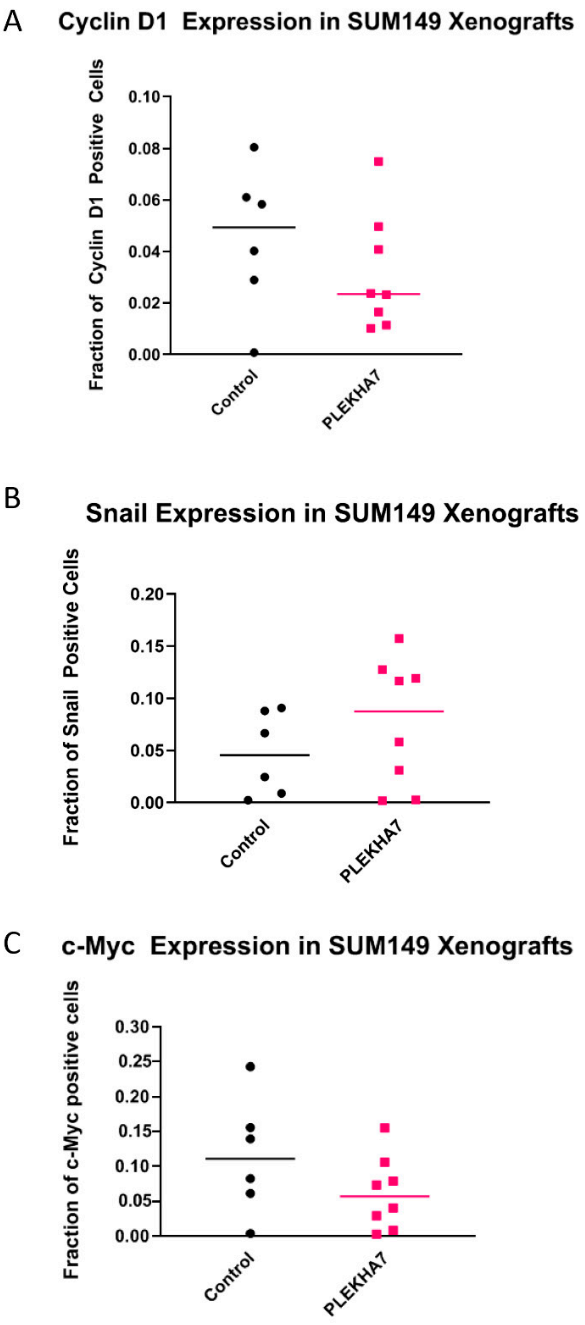

Figure S4. Expression of Cyclin-D1, Snail, and c-Myc in xenograft tumors. **A**) Fraction of Cyclin-D1 positive cells from SUM149 LZRS ms neo (control) or SUM149 LZRS PLEKHA7 xenografts at the 8-week end-point. **B**) Fraction of Snail positive cells from SUM149 LZRS ms neo or SUM149 LZRS PLEKHA7 xenografts at 8-weeks. **C**) Fraction of c-Myc positive cells from SUM149 LZRS ms neo or SUM149 LZRS PLEKHA7 xenografts at 8-weeks. *n*=6 for control group, *n*= 8 for PLEKHA7 group.

Table S1. Characterization of IBC Patient Samples.

Location	Solid/Tubular formation	Nuclear pleomorphism	% of SOLID tumor that is P7+	pattern for SOLID areas	% of overall tumor expressing basal PLEKHA7	% of P7 in gland	P7 pattern in gland	% of overall tumor that is apical PLEKHA7+	PLEKHA7 intensity
Dermis	97/3	2	90	basal	87.30%	90	apical	2.70%	3
Lymph node	99/1	3	70	basal	69.30%	50	apical	0.50%	2+3
Dermis	99/1	3	60	basal	59.40%	50	apical	0.50%	2+3
Lymph node	98/2	3	20	basal	19.60%	5	apical	0.10%	1
Breast	97/3	3	90	basal	87.30%	40	apical	1.20%	3
Dermis	100/0	3	50	basal	50%	no glands	none	0	2+3
Dermis	95/5	3	70	basal	66.50%	60	apical	3%	2+3
Dermis	95/5	3	60	basal	57%	0	none	0	2
Lymph node	90/10	3	90	basal	81%	50	apical	5%	2+3
Lymph node	98/2	3	70	cytoplasmic	68.6% (CYTOPLASMIC)	0	none	0	2
Breast	75/25		0		0	40	apical	10%	2
Breast	90/10	2	70	basal	63%	60	apical	6%	2+3
Dermis	60/40	3	50	basal	30%	50	apical	20%	2
Dermis	96/4	3	70	basal	67.20%	70	apical	2.80%	2+3
Dermis	96/4	3	10	basal	9.60%	0	none	0	2
Dermis	97/3	3	10	basal	9.70%	0	none	0	2
Dermis	97/3	3	10	basal	9.70%	0	none	0	2
Lymph node	95/5	3	80	basal	76%	30	apical	1.50%	3
Dermis	97/3	2	50	basal	48.50%	0	none	0	1
Dermis	99/1	2	90	cytoplasmic	89.1% (CYTOPLASMIC)	0	none	0	2
Dermsi	98/2	3	20	basal	19.60%	0	none	0	1
Lymph node	98/2	3	40	basal	39.20%	0	none	0	1+2
Breast	99/1	3	20	basal	19.80%	0	none	0	1+2
Dermis	75/25	3	0		0	80	apical	20%	3
Breast	80/20	3	70	basal	56%	30	apical	6%	3
Dermis	95/5	2	90	basal	85.50%	80	apical	4%	3
Dermis	100/0	2	40	basal	40%	0	none	0	2
Lymph node	90/10	3	0		0	10	apical	1%	2
Lymph node	85/15	1	0		0	0	none	0	1
Lymph node	95/5	3	50	basal	47.50%	40	apical	2%	2
Lymph node	95/5	2	90	basal	85.50%	80	apical	4%	3
Lymph node	90/10	3	30	basal	27%	30	apical	3%	2
Dermis	90/10	3	0		0	70	apical	7%	2
Dermis	100/0	3	10	basal	10%	0	none	0	1
Dermis	100/0	3	30	basal	30%	0	none	0	2

Breast	60/40	3	0		0	70	apical	28%	3
Breast	50/50	2	40	basal	20%	40	apical	20%	2+3
Breast	95/5	3	90	basal	85.50%	90	apical	4.50%	3
Breast	45/55	2	70	basal	31.50%	40	apical	22%	2+3
Breast	20/80	2	40	basal	8%	0	none	0	2+3
Breast	99/1	3	30	basal	29.70%	0	none	0	2
Breast	98/2	3	40	basal	39.20%	0	none	0	2
Breast	95/5	2	90	basal	85.50%	0	none	0	2
Breast	99/1	3	80	basal	79.20%	80	apical	0.80%	2+3
Breast	95/5	3	70	basal	66.50%	80	apical	4%	2+3
Breast	100/0	2	30	basal	30%	0	none	0	1+2
Breast	99/1	2	10	basal	9.90%	0	none	0	1
